# Feasibility of an Alcohol Intervention Programme for TB Patients with Alcohol Use Disorder (AUD) - A Qualitative Study from Chennai, South India

**DOI:** 10.1371/journal.pone.0027752

**Published:** 2011-11-21

**Authors:** Beena Thomas, Mohanarani Suhadev, Jamuna Mani, B. Gopala Ganapathy, Asaithambi Armugam, F. Faizunnisha, Mohanasundari Chelliah, Fraser Wares

**Affiliations:** 1 Tuberculosis Research Centre, Chetput, Chennai, Tamilnadu, India; 2 Office of the WHO Representative to India, New Delhi, India; McGill University, Canada

## Abstract

**Background:**

The negative influences of alcohol on TB management with regard to delays in seeking care as well as non compliance for treatment has been well documented. This study is part of a larger study on the prevalence of AUD (Alcohol Use Disorder) among TB patients which revealed that almost a quarter of TB patients who consumed alcohol could be classified as those who had AUD. However there is dearth of any effective alcohol intervention programme for TB patients with Alcohol Use Disorder (AUD).

**Methodology:**

This qualitative study using the ecological system model was done to gain insights into the perceived effect of alcohol use on TB treatment and perceived necessity of an intervention programme for TB patients with AUD. We used purposive sampling to select 44 men from 73 TB patients with an AUDIT score >8. Focus group discussions (FGDs) and interviews were conducted with TB patients with AUD, their family members and health providers.

**Results:**

TB patients with AUD report excessive alcohol intake as one of the reasons for their vulnerability for TB. Peer pressure has been reported by many as the main reason for alcohol consumption. The influences of alcohol use on TB treatment has been elaborated especially with regard to the fears around the adverse effects of alcohol on TB drugs and the fear of being reprimanded by health providers. The need for alcohol intervention programs was expressed by the TB patients, their families and health providers. Suggestions for the intervention programmes included individual and group sessions, involvement of family members, audiovisual aids and the importance of sensitization by health staff.

**Conclusions:**

The findings call for urgent need based interventions which need to be pilot tested with a randomized control trial to bring out a model intervention programme for TB patients with AUD.

## Introduction

Alcohol use- disorders (AUD) has been reported as a risk factor for an impaired immune system, and increases a person's susceptibility to active TB infection as well as to the reactivation of latent disease [1]–[8]. While 40% of the Indian population is infected with the TB bacillus, only about 10% develop the disease [9]. There are many factors that contribute to developing TB and based on the assumptions of Lonnrot and colleagues [4], the proportion of TB attributable to alcohol as a risk factor in different countries is substantial. India is no exception with the challenge of poverty, high rates of alcohol use and hazardous drinking among men [10]. This not only increases vulnerability to TB but there are other issues of concern which vary from delays in diagnosis [11] to default for treatment and mortality [12], [13]. Furthermore AUD and TB have both been labeled as “diseases of poverty”, and both can be consequences as well as causes of social marginalization [14]–[16]. The TB clinics do not regularly screen patients for AUD and the information available in the case records of TB patients is limited to whether they consume alcohol or not. This study is part of a larger study to document the prevalence of alcohol abuse among TB patients using an AUDIT (Alcohol Use Disorder Identification Test, WHO, 2001). The study reported 29% of alcohol consumption among TB patients with 52%(73) of them having a score of >8 in the AUDIT scale [17]. According to the AUDIT scale those who scored above 8 are referred to as hazardous, harmful and dependant drinkers. The study highlighted that the treatment outcome was unsatisfactory for more than a tenth of those patients with AUD and hazardous drinking leading to chronic treatment default, treatment failure and death. Treatment failures were 43% in the low risk group with AUDIT score <8 against 57% in group of TB patients with AUDIT score >8. This qualitative study was done to describe the reasons for excessive alcohol intake, the problems associated with alcohol abuse, the perceived effect of alcohol use on TB treatment and the perceived necessity of an intervention programme for TB patients with AUD. The findings of the study would help evolve effective intervention strategies for TB patients with AUD.

### Conceptual framework for the study

The framework adopted for the study was the Ecological Systems model, introduced by Bronfenbrenner (1979). The Ecological framework was helpful in understanding the underlying influences of AUD and perceived effect on TB management.

#### Micro system

Refers to the closest influences such as peers, family, workplace and neighborhood. Here the influences are bi-directional (both away and towards the TB patient with AUD). They contribute to the individual taking alcohol (peer pressure, low self esteem, problems within the family. occupational influences). Furthermore individual behaviour due to AUD has an influence on these factors (problems within the family, gaining acceptance from peers, ability to work)

#### Meso system

The interconnections between the structures of the micro system and the health environment (alcohol use and vulnerability to TB)

#### Exo system

The larger environment: the availability and accessibility of alcohol which influence the alcohol behavior. (Liquor shops situated near TB clinics)

#### Macro system

This comprises of cultural values such as negative consequences (Social disapproval, labeling, stigma, social exclusion) for those who engage in excessive drinking.

#### Chrono system

Encompasses the dimension of time as it relates to the patients environment such as patients perceptions of the influence of alcohol on TB treatment (adverse effects of drugs and alcohol fear of being reprimanded during the treatment period)) which could determine his treatment compliance.

## Methods

A qualitative approach utilizing focus group discussions (FGDs) and interviews were conducted with TB patients with AUD, health providers and family members of TB patients with AUD.

### Setting and Participants

The study was conducted in urban Chennai in 4 Corporation zones out of the 10 corporation zones offering TB treatment. The zones were randomly selected based on the number of TB patients registered during July to September 2009.

Participants were TB patients who completed an AUDIT [18] screening tool, had a score of >8, conversant in Tamil and willing to give consent and spend time for the FGDs and interviews. Overall out of the 490 TB patients screened, 141 TB patients reported alcohol consumption and 73 of them had an AUDIT score of >8, [17]. We used purposive sampling to select 48 TB patients (12 from each zone) from the 73 TB patients with AUD for the FGDs and the interviews.

**Figure 1:**
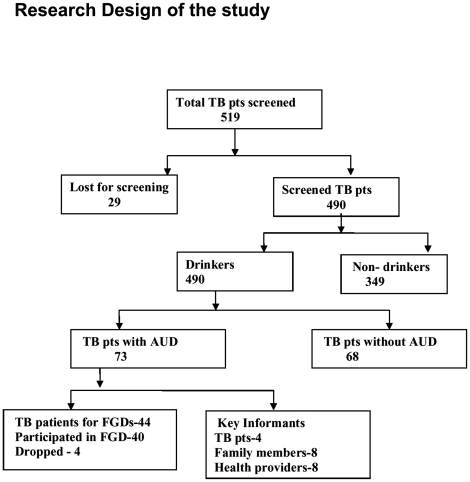
Research design of the study.

The 48 patients were screened by the counselors of which 44 were willing and their consent was obtained and they were enrolled for the FGD and 4 for the interviews. The date of the discussions and interviews were decided based on their convenience and the venue was the clinic they attended to collect their drugs. However 4 participants dropped out for the focus group discussions and only 40 attended the FGDs. All 4 TB patients attended for the interviews. (Figure 1)

Apart from TB patients a convenient sample of 8 health providers (2 from each zone) and 8 family members of TB patients with AUD (2 from each zone) were considered for the interviews. The family members were the spouses or a parent of the TB patients with AUD and the health providers were clinicians, treatment supervisors and health visitors.

### Study Instruments

The semi-structured qualitative FGD and interview guides were based on a literature review and previously conducted qualitative information gathered by the team during the situational analysis before the start of the study. They were pilot tested among 2 TB patients with AUD and one FGD with 5 TB patients with AUD. These patients were not included in the interviews or FGDs done as part of the study. The guides were translated into Tamil and back-translated into English to ensure accuracy.

The topic areas in the guides and the interviews were on perceptions and reasons for excessive alcohol use, perceived effect of alcohol use on family, self, and society, perceptions on TB and alcohol consumption during TB treatment. Information on the need and suggestions for alcohol intervention measures during TB treatment were also included. The interviews with the health providers and families broadly addressed the challenges in dealing with TB patients who excessively consumed alcohol, their concerns, need for intervention and possible intervention areas. (Table 1)

**Table 1:** Sample questions.

Sample questions for FGDs and Interviews
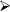 Do you think TB is curable? 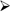 What do you think are the reasons for you drinking alcohol? 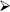 Do you think alcohol use has anything to do with TB? 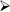 In your opinion does alcohol use affect TB treatment compliance? 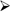 What do you perceive as the problems you encounter because of alcohol use? 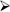 Could you elaborate on reasons why you might have missed your TB medication because of alcohol use? 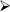 Have you experienced any discrimination on account of the alcohol use? 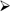 Do you perceive the need for alcohol intervention programs in TB clinics? 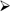 What might be some of the intervention strategies that would be acceptable and feasible?

A team of 6 trained counsellors (3 men and 3 women) who were involved in the larger study to estimate the prevalence of AUD were involved in this study. Their efforts included screening of participants for the study and were involved in the conduct of the interviews and FGDs. All of them had a Masters degree in Social Work(MSW) had 2 years expertise in dealing with socio-behavioural and gender issues both in TB and HIV. They also underwent training in qualitative research and in using the study instruments. Furthermore their involvement in the larger study helped since they had already established a good rapport with the TB patients and providers. The same counsellors who did the initial AUDIT in the 4 zones were assigned to carry out the FGDS and the interviews in the respective zones.

The setting of the FGDs and the interviews were in the TB clinics in a private room that helped maintain privacy and confidentiality. This venue was chosen based on the convenience of the participants. They were encouraged to share their experiences freely and were assured privacy and confidentiality. A facilitator conducted the FGDs with the help of a note taker. Eight focus groups were conducted with 40 study participants. Each FGD lasted for 60 to 75 minutes, sometimes it led to 1 hour 30 minute. These would normally be considered lengthy discussions, but this situation resulted from the desire of the participants themselves to talk freely and at length about their experiences. Care was taken to see that all the participants contributed to the discussions with the help of a sociogram prepared by the note taker during the discussion.

The individual interviews (one to one) lasted for 30 minutes to 45 minutes for the TB patients with AUD. They were done by both the male and the female investigators as the patients (all males) expressed that they were comfortable with both the male and female investigators. The interviews for the health providers and family members took an average of 20 to 30 minutes.

Most of the respondents in the FGDs and the interviews (44) were in the age group between 21years to 50 years. Two thirds of them were married and except for 3 respondents, who had no formal education, the others had some degree of education with the majority having primary or middle school education. Two thirds of the respondents were married and those who were single lived with their parents. While half of the respondents were daily wage earners, nearly one third had monthly salaried jobs.

Each interview and focus group was digitally recorded. The digital recordings were transcribed verbatim by the study investigators and then translated into English for the purposes of analysis. The team regularly met to discuss emerging themes and issues as well as to minimize bias caused by differential interviewer methods.

The overall design included a strong focus on participatory research that sought the contributions of the participants in its design and implementation [19].

### Ethics Statement

The study was approved by the Scientific Advisory Committee and Institutional Review Board of the Tuberculosis Research Centre (Indian Council of Medical Research) and written informed consent obtained for all study participants.

### Data analysis

Data was analyzed using thematic content analysis guided by a grounded theory methodology. This allowed core themes to arise from the data. Analysis focused on themes that were relevant to the focus areas in framework adopted for the study. Transcripts were reviewed for errors and omissions including context and content accuracy The qualitative analysis was done using the MAX qda software to code and thematically organize the transcripts.

## Results

Transcripts from the original data transcribed from the FGDs and interviews with TB patients with AUD were divided into 6 broad themes which included 1) Perceptions of excessive alcohol consumption 2) Reasons for alcohol use and perceptions of alcohol as a disease 3) Impact of excessive intake of alcohol on family, society and self 4) Perceptions on TB 5) Perception on Alcoholic Consumption during TB Treatment 6) Perceptions on alcohol intervention programs. **Table 2** elaborates the themes and the key findings.

**Table 2:** Themes and Key findings.

Themes	Key findings
Perceptions of TB patient's excessive alcohol consumption	• When a person starts drinking first thing in the morningDrinks large amounts of alcohol everyday from quarter bottle (180 ml) to 3 bottles per day, Abstains from work, Wakes up at night and has alcohol.
Reasons for alcohol use	• Peer pressure,• Starts as an occasional drink at parties,• Physical pain, family problems, work pressure,
Influence of excessive intake of alcohol on self family society	• Affects health,• Angry reactions, affects relationships within families• Financial instability• Low self esteem• Discrimination, labeling, exclusion from social events
Perceptions on Alcohol use and TB treatment	• Alcohol use increases vulnerability for TB• Reduces therapeutic effect of TB medication,• Reason for default due to fear of adverse side effects of drugs due to alcohol• Fear of being reprimanded by health staff when reporting to the clinic smelling of alcohol.• Inability to control the craving for alcohol
Perceptions on intervention for TB patients who consume alcohol excessively.	• Lack of alcohol intervention in TB clinics• Need for alcohol intervention• Suggestions included: motivation from clinicians,• audio-visuals, individual & group sessions,• family members to be involved in intervention and• integration of alcohol de addiction services in TB clinics• Removal of liquor shops near TB clincs
Perceptions on alcohol intervention programs from health care providers and family members of TB patients	• Need for alcohol intervention programsClinicians and health staff need to be trained and equipped to screen and deal with TB patients with AUD.

The transcripts of the interviews done for the health providers and family members have been presented separately. These interviews focused primarily on their perceptions of an alcohol intervention programme for TB patients with AUD.

### 1. Perceptions of TB patient's excessive alcohol consumption

Drinks that were commonly reported by the participants in the FGDs and the interviews were brandy, whisky and beer. Few reported using arrack (locally made), varnish, thinners (polishing materials) or whiteners (chemical) used for inhaling purpose. Beer according to the majority was the foundation drink which later changed to harder forms of alcohol. Most of the respondents in the FGDs said they had quarter bottle (180 ml) of either whisky or brandy a day, and few said they drank even up three quarters of a bottle of brandy or whisky and one respondent during the interview said he had two bottles of brandy a day.

Most of the respondents in the FGDs and interviews said that excessive drinking was when a person starts drinking first thing in the morning, even before he brushed his teeth. One respondent during the interview said that excessive drinking of alcohol was when a person woke up at night restless to have a drink and one said every time he got up at night he would drink alcohol. Some of the respondents in the discussions said that those who drank excessively had alcohol placed close to where they slept. Many opined that excessive drinkers were those who drank alcohol during the day preventing them from going to work. They would somehow gain access to alcohol. They also commented on the unsteady gait of excessive drinkers, which makes them so visible. Few of the respondents in the FGDs and two during the interviews said that excessive drinkers were those who did not eat properly when they consumed alcohol and were prone to many diseases.

***“There are some men who cannot even work without a drink. Even if the shops were closed they would somehow procure it.”***

***“You can make out those who drink excessively as they have an unsteady gait and are prone to fall down on the road.”***

A number of the respondents in the FGDs and all those in the interviews reported excessive drinkers as those who consumed large amounts of alcohol each day from quarter bottle to even 3 bottles per day. An interesting terminology that came out from the discussion was the term used by one of the respondents “King of alcoholics” referring to those people who drank alcohol from dawn till night. The others agreed with him.

### 2 Reasons for alcohol use, perceptions on alcohol as a disease

Many of the respondents in the FGDs said peer pressure especially in their adolescent years was the main reason for their dependency on alcohol. The general feeling among them was wanting to be accepted by their peers when they drank alcohol or avoiding being made fun of or being called names reflecting their weakness. This was strongly felt by three of the respondents during the interview.

Most of the respondents in the interviews as well as the FGDs stated that it started as an occasional drink at parties or during festival times for fun which later became a habit. Many of the men said that they indulged in alcohol to cope with family problems, while others to work pressure. A few of them during the FGDs and two of them during the interview explained that they indulged in alcohol to forget their physical pain or discomfort.

***“Everyone starts off drinking with a bottle of beer with friends. Then as we grow up, we shift to brandy or whisky. It starts for fun and then it becomes a coping mechanism. Now every time there are problems in the family, I drink”***

***“It is difficult to stop drinking. I am unable to sleep without drinking”***

Alcohol dependency leading to diseases was felt by many respondents in the FGDs as they felt that alcohol affected the gastrointestinal tract, TB and could cause many associated problems. Only one respondent talked about alcohol leading to stomach ulcers.

Few however said that while it could not be considered as a disease as unlike TB there was no treatment for alcohol dependency.

Most of the respondents however said that it depended on the individual to give up excessive drinking of alcohol and it was not a disease that required treatment.

***“It totally depends on the individual to choose to give up the habit and this could only be achieved through self control”.***

Few in them in a group shared that religion also played a role in quitting the alcohol habit. Excessive alcohol leading to risky sexual behavior both premarital and extra marital sexual relationships was also expressed by the participants.

### 3. The impact of excessive alcohol use on family, society and self

#### Family

Some of the respondents said that the impact was more financial resulting in their inability to maintain their family.

***“Due to my alcohol dependency I had a lot of financial problems and I have not been able to play the role of husband or be responsible”***

“I seem to be always in debt. There never seems to be enough money. This leads to frequent problems at home”.

Furthermore many respondents reported that under the influence of alcohol tempers soared high and that they did not even remember what happened when they drank too much alcohol. This led to frequent quarrels within the family leading to discord in relationships with their spouse and children.

***“I have frequent quarrels with my wife and children when I am drunk.***

***Because of these fights our sexual life is affected”.***

***“Because of my behavior I am spoiling the whole peace in the family but I cannot control this”.***

Another problem often expressed by many the respondents in the FGDs and three in the interviews was their insecurities in relationships.

***“Since I drink so much my friends and neighbors suspect me whenever I contact them. They are always worried that I will ask them for money”.***

#### Society

Most of the respondents in the FGDs and all during the interviews expressed problems with regard to discriminatory treatment from relatives and friends who disrespect them because of their drinking habit. They felt that they were labeled as drunkards and excluded from social functions.

***“I am not even invited for a social function. I am labeled as a kudikaran (drunkard) and even if I am invited they ignore me at the function. They do not even listen to me if I speak and even if I say anything they do not take it seriously”.***

***“I find it difficult to get any help from anybody. Even when I need money nobody would give me. Even if I do give up drinking I doubt anybody would believe me. Even if there is a genuine reason and I raise my voice at home, neighbors immediately conclude that I am drunk”.***

#### Self

Several of the participants both during the FGDs and all during the interviews said they were aware that excessive drinking was harmful. Some said that it could lead to physical damage such as liver damage, gastrointestinal TB, ulcers and memory loss. Other responses were the severe after effects they faced after a bout of drinking which burdened them with frequent headaches and loss of memory. One respondent in interview said that under the influence of alcohol he found it difficult to ride his bike and has fallen several times and had injuries.

***“I have often met with minor accidents on my two wheeler after I have had too much to drink”.***

Few of them in the FGDs and in the interview said that alcohol caused dizziness the next day when they woke up preventing them from attending work. Some of the participants said that too much drinking could also lead to cancer and blood vomiting. Memory loss was often quoted after a drinking episode.

Few of the respondents shared that they always felt guilty after consuming alcohol excessively. They reported low self esteem especially when they faced disrespect from their family members.

***“My wife suspects me all the time. Even if money is misplaced, lost or stolen by some one else at home,I am blamed.. Even when I speak the truth they don't trust me.”***

### Perceptions on TB

A common response among the men in the discussions and interviews was that alcohol was the main cause for their diagnosis of TB. While many of them perceived TB to be an air borne disease, they also opined that their intake of alcohol had increased their vulnerability to getting TB. Two of them during the interview said that drinking lowered their appetite and attributed lack of a nutritious diet to their diagnosis of TB.

***“People who drink excessively are thrown out of the house, do not eat; sleep in the open and are exposed to the cold and are prone to many diseases”.***

Some attributed the spread of TB to stepping on the sputum or saliva of an infected person. Some respondents during the FGDs and two during the interview said that staying away from TB infected persons was one way of protecting themselves from TB. Other responses were that TB infected persons had to avoid indiscriminate spitting on the road and had to be cautious in covering their mouth while coughing.

### Alcohol among women

Many during the FGDS and 2 during the interviews said that it was uncommon for women in the lower socioeconomic strata to drink. They felt that men were more exposed to alcohol use due to pressure from their friends and due to the nature of their work. Few also said that acceptance by women that they drank alcohol was uncommon. However two of them during the discussions disagreed and said drinking alcohol was more common among the upper class women, although it was usually hidden.

Two of them during the interviews said that husbands themselves introduced their wives to alcohol, as a way to cope with stress and fatigue. Other responses from participants during the interviews was that women who did illegal things (sex, drugs) consumed alcohol and also sent their own children to buy alcohol for them.

### Perception on Alcoholic Consumption during TB Treatment

Many respondents during the discussions and the interviews felt that drinking alcohol while on treatment could reduce the therapeutic effect of their TB medications. Some of the respondents also said that they could not help drinking during TB treatment but chose not to turn up for treatment on days when they have had drinks as they are worried about adverse effects of medicines taken with alcohol. Two respondents during the interview said that they suffered stomach upsets when they combined alcohol with their TB drugs.

***“I worry if the alcohol would mix badly with the TB drugs.”***

***“I was told by the doctors that if I take alcohol during TB treatment I will cough out blood.”***

***“I am scared of going to the TB clinic after I have had alcohol as I am sure I will be shouted at when they smell alcohol on me”.***

***“When I drink alcohol and consume TB medicine simultaneously there are side effects like drowsiness, vomitingand my stomach aches. I am not able to manage these side effects of TB treatment and alcohol”.***

Some of the respondents also reported that they avoided going to the TB clinic after an episode of drinking alcohol the previous evening, as they feared being reprimanded by the clinicians and other staff when they attended smelling of alcohol. This was also expressed by three of the respondents during the interviews.

***“It is very embarrassing attending the clinic smelling of alcohol. The smell stays on”.***

***“I avoid attending as I fear I will be shouted at by the staff in the clinics. Everybody then knows I have taken alcohol”.***

The inability to avoid alcohol was expressed by many during the FGDs and 3 during the interviews. This has also been one of the reasons for non compliance for TB treatment.

***“I am not able to control craving for alcohol so I have missed treatment several days”***

***“I usually have a drink on non treatment days but sometimes I get tempted to drink on treatment days as well. In this case I avoid taking the TB drugs. This has happened on many occasions and now I have decided to give up drinking till I complete treatment”.***

There were some who perceived that a disease like TB was enough to give up alcohol.

***“Ever since I was diagnosed with TB, I quit alcohol”***

***“When I brought out blood stained sputum, I got so scared, I stopped drinking”***

Perceptions and suggestions on intervention for TB patients who consume alcohol excessively.

The majority of the respondents felt that intervention programmes for TB patients who had alcohol related problems were lacking. Many respondents both in the discussions as well as the interviews said it is important to have an intervention programs for TB patients who were dependant on alcohol as this would help them to cope with problems related to alcohol abuse, default for TB and the fears associated with alcohol dependence and TB treatment.

**“An *alcohol intervention programme would benefit those of us who cannot help taking take alcohol during TB treatment”.***

They expressed the fact that this was not being done in any health care facility. There were also few who reported that the staff in the TB clinics could offer services for alcohol dependant patients when they were enrolled for TB treatment. Suggestions included motivation by clinicians, group sessions and audio-visuals on the adverse effects of alcohol abuse and TB in clinics. This included screening of short films in TB clinics while they wait for treatment as a general way of communicating messages on alcohol abuse and TB management. Suggestions also included posters and pamphlets with simple, clear messages on the dangers of alcohol abuse and TB treatment. One of the respondents during the FGD remarked that the accessibility of liquor shops in front of the TB clinics was a big temptation especially when their symptoms subsided and the desire for alcohol surfaced. This was agreed by all and they all felt efforts should be taken by the government to remove these shops in front of TB clinics.

***“How do you expect TB patients like us who have a drinking problem to avoid it when you have these TASMAC (liquor) shops right in front of the TB clinic”??***

***“It is important for someone to take visuals of what happens in the causality department of hospitals where people under the influence of alcohol are brought in after an accident profusely bleeding or vomiting blood. Maybe that shock would help people into giving up alcohol”***

***“Nobody talks to us about the dangers of alcohol. We are reprimanded for taking alcohol but there is no intervention program for TB patients who consume alcohol”***

***“Health clinics have posters, visuals on HIV, maternal care but nothing on alcohol. It would be helpful for patients to be made aware of the dangers of alcohol in all TB clinics”***

Their perceptions on the preference of individual or group sessions for intervention were also ascertained. Most of the participants during the discussions and interviews felt that both were important. However few opined that individual sessions would be useful only when the patient was willing to have a session as many would not like to be singled out. Many felt that the clinicians should include the dangers of alcohol abuse when he dealt with all patients irrespective of whether he had an alcohol problem.

**“It *would be good for doctors to tell all TB patients about the ill effects of alcohol when they start them on treatment. Even if they occasionally drink let them know”.***

***“It depends on the TB patient whether he will be willing to have an individual session with the health staff. They may not want others to know that they consume alcohol”.***

Many both during the discussions and interviews said they were comfortable with support group sessions where patients who had successfully given up alcohol abuse could come and share with others their experiences. Some were aware of the Alcoholic Anonymous group for alcoholics but had not participated in any of their programs.

***“Those patients who already completed treatment successfully but had alcohol dependency could come and share their experience of how they followed abstinence from the alcohol during their treatment phase”***

Participants expressed the need for family members to be included in the intervention programme as their support was necessary in helping alcoholic patients to deal with the problems related to alcohol abuse. This was expressed by three of the respondents during the interviews.

***“It is important for spouses to be involved in an intervention program as they would understand us better and also help us complete our TB treatment by motivating us at home. It is important for continuous reminders”.***

***“We should involve the family members because they are the one who are being with us for more hours a day. So definitely we should make them involved. Family members should check the patient about his regularity towards treatment”***

However a few respondents during the discussions cautioned against being forced to quit alcohol especially from their wives which they deeply opposed.

***“It is difficult when spouse coerce us to give up the alcohol habit. That is what makes us want to continue drinking”***

Most of the respondents were open to de addiction services but were confused as de-addiction centers were too expensive but offered cure within 3–4 months. Suggestions included that these services be available in the TB health care facility to facilitate better accessibility, monitoring and promote adherence to TB drugs. Many also opined that this would facilitate sustainability even when the treatment for TB was completed. Importance of family and social support system to deal with alcohol abuse was widely felt.

***“A disease like TB is enough to shake you out of alcohol use as you are so afraid of the consequences. When you start feeling better with TB treatment and you want to give up alcohol, you see a huge liquor shop in front of the hospital. How does society help us overcome this temptation?”***

### Perceptions on alcohol intervention programs from health care providers and family members of TB patients

All the health providers interviewed said that they were not trained in screening TB patients for AUD and had no experience in any alcohol intervention program for these patients. While most of the clinicians said they advice patients on the dangers of alcohol if they report alcohol use, they did not have any structured intervention in place. Many felt that alcohol did interfere with TB management as they defaulted when the symptoms were better. The treatment supervisors expressed helplessness in dealing with TB patients who were alcohol dependant. An alcohol intervention programme for TB patients was felt by all the heath providers but apprehensions as to how it should be done and if it could be done in the clinics was expressed by all.

***“We tell our TB patients on treatment not to drink while on treatment. We do not screen them for alcohol abuse. We go by what the patient or his family says”. (Medical officer- TB clinic)***

***“Alcohol is a problem in managing TB patients as once symptoms disappear, they get back to it. We can only tell them to be responsible and complete their treatment”. (Medial officer- TB clinic)***

***“We have not been trained to deal with TB patients who are alcohol dependent. We go to their houses if they do not come for TB drugs and we tell them about the dangers of alcohol. They do not seem to care. I do not know if those who are addicted to alcohol can ever be helped”. (Treatment supervisor)***

***It is so frustrating when they do not keep up their word. They say they will not touch alcohol but once they feel better, they go back to alcohol”. (Outreach worker-TB clinic)***

***“It is important to have an alcohol intervention programme for TB patients with alcohol dependency but we need to know what works best. At present there is no intervention programme in place”. (Treatment supervisor)***

Family members opined on the urgent need for intervention programs for those with AUD and felt that this would help to facilitate treatment completion as well as a better quality of life or themselves and their children.

***“It is good that my husband has given up drinking but that may be short lived as he has been drinking all his life. It is important to scare him on the dangers of alcohol lest he starts again” (Wife of a TB patient)***

***“It is important that the doctors, nurses, the staff who come home keep reminding TB patients of the dangers of alcohol every time they see the patient. They need to be constantly reminded as once they feel better they are tempted to take alcohol” (Mother of a TB patient)***

Many of the family members felt that they could be included as their involvement would help while there were opinions on their difficulties in being part of such a programme. While some were optimistic of the benefits of an intervention programme and few were convinced that it was difficult for alcohol dependent persons to come out of their addiction.

## Discussion

The findings of this qualitative study bring out the problems of alcohol use disorder among TB patients which poses a challenge to the TB control programme. Peer pressure has been a strong influence for alcohol abuse, especially among young men. This highlights the need for alcohol intervention at different levels. This is corroborated with studies that have reported that India's alcohol and drug use rates are rising, among adolescents [20]–[22]. This could suggest that alcohol intervention at an early age could bring down the reported risks associated in terms of vulnerability to the TB disease [Bibr pone.0027752-Szabo1] [3&4].

Alcohol use among men infected with TB is reported more than women and the perception that men are more vulnerable to TB seem to be common in the community [Bibr pone.0027752-Ganapathy1] [23&24]. This could be partly true considering that men are more exposed to alcohol abuse with their social contacts and occupations and that woman face cultural restraints that could inhibit their acceptance to even taking alcohol. It also important to take note of the perceptions within the community where TB patients face stigma related to the disease [Bibr pone.0027752-Jaggarajamma1] [25 &26]. This study has also brought out the stigma that TB patients face with regard to their excessive alcohol use. This could result in a dual stigma of having TB and AUD which needs to be addressed in alcohol TB intervention programs.

A recent study showed that among patients with TB who interrupted the course of treatment, 47.7% were heavy drinkers. The odds ratio for an interruption of treatment was 3.8 for heavy drinkers compared to other patients [27]–[29]. Furthermore alcohol dependence together with social exclusion leads to delays in seeking and starting the treatment of TB and worsening its course in that way [30].

This study provides insight on the problems associated with adherence for TB treatment among TB patients who consume alcohol excessively. The reasons for non adherence for TB treatment are the fears associated with taking TB drugs after taking alcohol. These fears are varied. The fear of the adverse effects of TB drugs and alcohol, the fear of the reduction in the therapeutic effect of the drugs when alcohol is consumed, and the fears of being reprimanded by health providers when they report to the clinic with an odor of alcohol. The need for health providers to be sensitized on these issues is crucial as motivations strategies should be skillfully done to facilitate patients to take their TB drugs This could be further explained as one respondent expressed “The doctor told me that I need to abstain from alcohol when I take TB drugs as there are more chances that I would bring out blood when I cough if I take alcohol with TB drugs. I therefore avoided my drugs even if I have one drink during a social function.”

Family members of patients with AUD have expressed the urgency to have alcohol intervention programmes for TB patients who have alcohol related problems. This also reflects their concern that an intervention program had to be in place in a way reflecting their helplessness in dealing with the problems around AUD and TB treatment. It is also important for family members be involved in the TB intervention programs as TB patients with AUD have often expressed negative reactions and stigma from family members due to their alcohol use. These reactions could be worse once the patient is diagnosed with TB which could be a deterrent to patients TB treatment compliance.

Health providers report lack of training in screening for AUD and there is no intervention in place for TB patients with AUD. This endorses the need for alcohol screening and feasible intervention programmes in health facilities offering TB management. A study from Russia reported successful introduction of the AUDIT to screen TB patients for alcohol use disorder, established referral algorithms, and hired additional staff to integrate AUDs management in TB services. The study also reports that referral services for alcohol use disorders improved when TB physicians explained the effect of alcohol consumption on TB outcomes and framed alcohol management as an integral component of TB treatment [31].

An important aspect of this study is the perceptions of the TB patients with AUD on feasible interventions which would be beneficial to them. This included audio-visual aids, in clinics, individual and group sessions. The importance of families in the intervention programme was stressed especially in promoting adherence for TB treatment to a great extent. Health education and advocacy at all levels within families and communities is therefore crucial. It may range from something as simple as ensuring awareness regarding the disease, symptoms and treatment, to the simple act of putting up messages in outdoor clinics and using the mass media such as radio and television which have a powerful influence in the community.

### Strengths and Limitations

A potential limitation to the present study includes the fact that it is a small, purposive sample composed of TB patients with AUD from urban chennai willing to be interviewed or participate in a focus group, so generalizability is limited. Social desirability bias especially with regard to TB treatment compliance and excessive alcohol use could have limited responses. The strengths of this study are that all focus groups and interviews were conducted by trained investigators with adequate years of facilitation experience. This study has documented the concerns and suggestions of TB patients, their families and health providers in the TB clinics on effective alcohol intervention programs in TB clinics in the future.

### Conclusion

The study has provided a background for designing and testing an intervention programme that could be introduced in the health facilities. While early intervention is crucial in terms of vulnerability, the study highlights the need for careful and sustained intervention in all TB clinics at all levels from treatment initiation to treatment management and follow up.
